# The Value of Decreased Thyroid Hormone for Predicting Mortality in Adult Septic Patients: A Systematic Review and Meta-Analysis

**DOI:** 10.1038/s41598-018-32543-7

**Published:** 2018-09-20

**Authors:** Jae Guk Kim, Hyungoo Shin, Wonhee Kim, Tae Ho Lim, Bohyoung Jang, Youngsuk Cho, Kyu-Sun Choi, Chiwon Ahn, Juncheol Lee, Min Kyun Na

**Affiliations:** 1 Department of Emergency Medicine, College of Medicine, Hallym University, Chuncheon, Republic of Korea; 2 Department of Emergency Medicine, College of Medicine, Hanyang University, Seoul, Korea; 3 Department of Biomedical Engineering, Graduate School of Medicine, Hanyang University, Seoul, Korea; 4 Department of Neurosurgery, College of Medicine, Hanyang University, Seoul, Korea; 5 Department of Preventive Medicine, College of Korean Medicine, Kyung Hee University, Seoul, Korea; 6 Department of Emergency Medicine, Armed Forces Yangju Hospital, Yangju, Korea

## Abstract

Decreased thyroid hormone (TH) has been considered as one of the potential predictors of mortality in sepsis. This study aimed to evaluate the prognostic impact of decreased TH on mortality in septic patients during intensive care unit (ICU) admission. We included studies that assessed thyroid function by measuring the serum thyroid hormone level and in-hospital mortality in adult septic patients. Reviews, case reports, editorials, letters, commentaries, animal studies, duplicate studies, and studies with irrelevant populations and inappropriate controls were excluded. A total of 1,578 patients from eight studies were included. Triiodothyronine levels in non-survivors were relatively lower than that of survivors (6 studies; standardized mean difference [SMD] 2.31; 95% confidence interval (CI), 0.52–4.10; I^2^ = 97%; P = 0.01). Thyroxine levels in non-survivors were also lower than that of survivors (5 studies; SMD 2.40; 95% CI, 0.91–3.89). There were no statistically significant differences in thyroid-stimulating hormone levels between non-survivors and survivors. The present meta-analysis suggested that the decreased TH during ICU admission might be associated with the increase in mortality in adult septic patients. Hence, the measurement of TH could provide prognostic information on mortality in adult septic patients.

## Introduction

Decreased thyroid hormone (TH) level has often been reported in septic patients. This phenomenon has been explained by the key role of TH in adapting metabolic function to sepsis^1,2^. The two mechanisms which result in low triiodothyronine (T3) and thyroxine (T4) in sepsis are (1) the excessive production of inactive reverse T3 from T4 and (2) the malfunction of hypothalamic-pituitary-thyroid axis by inflammatory cytokine^3,4^.

The decrease in TH level is closely related to the dysfunction of immune cells or the heart muscles^5^. Furthermore, decreased T3 level is also correlated with worsening lung function^6,7^. In an animal study on sepsis, a decrease in free T3 (fT3) and free T4 (fT4) levels was observed in the septic rat group with histopathological damages of the liver, lung, and kidney^8^. These significant correlations between TH level and multiple organ dysfunction suggest that decreased TH level in sepsis could provide the prognostic information on mortality.

Previous clinical studies also demonstrated the association of the decrease in various TH levels such as T3, T4, thyroid stimulating hormone (TSH), fT3, and fT4 with mortality in septic patients^2,9–17^. As a result, the decrease in TH level has been considered as one of the potential predictors of mortality in septic patients^2,15–17^. Nevertheless, the prognostic impact of TH in mortality due to sepsis is still controversial.

We performed a systematic review and meta-analysis to identify the prognostic value of decreased TH levels in mortality due to sepsis by classifying TH types.

## Results

### Study and Patients Characteristics

**Figure 1:**
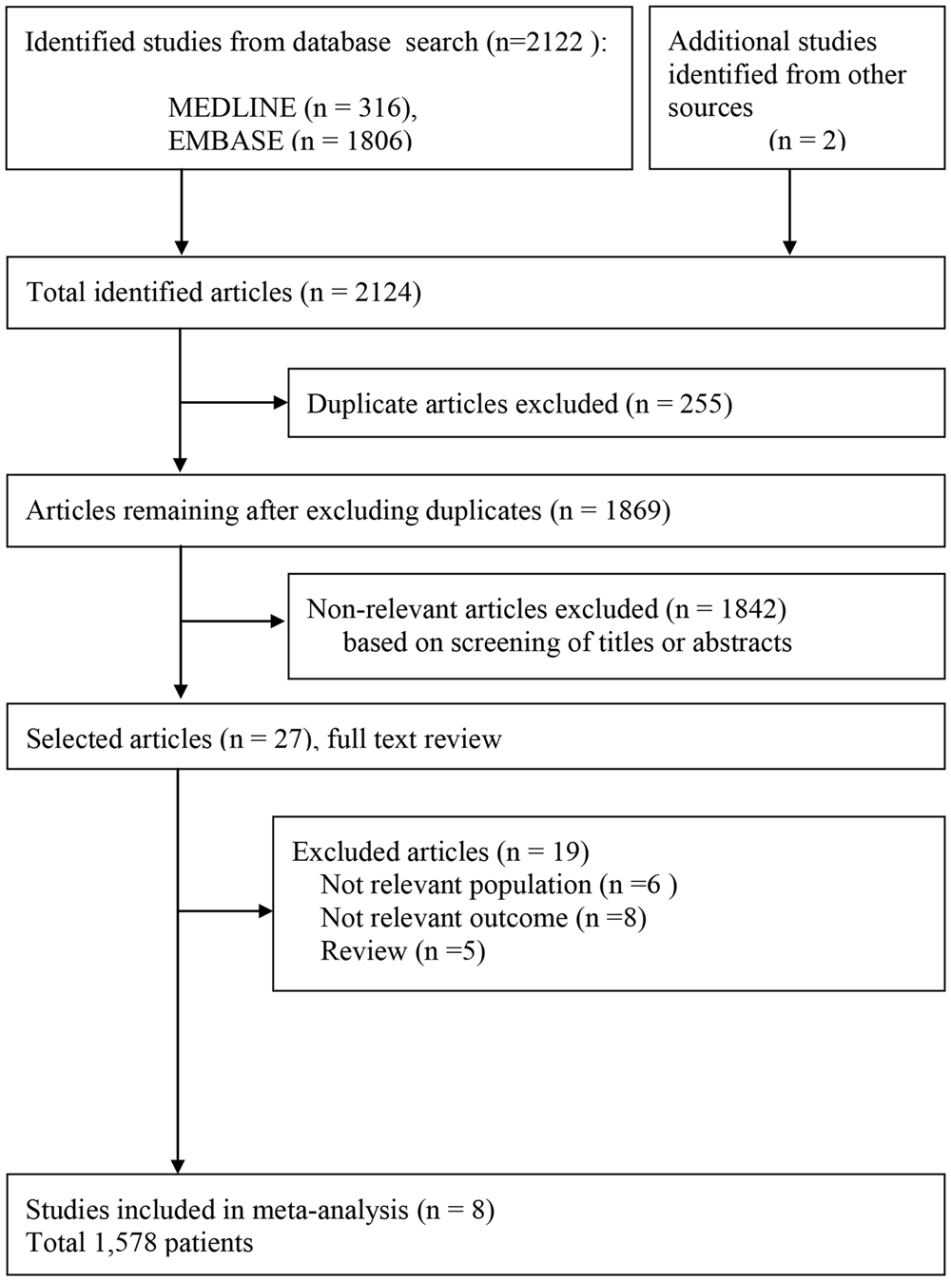
Flow diagram for identification of relevant studies.

The flowchart of the search and selection of eligible studies, is shown in Fig. 1. A total of 2,124 records were identified through database searching while two additional records were identified through other sources. After removing 255 duplicates, the titles and abstracts for 1,869 records were screened for eligibility. Of these, 27 records were identified as being potentially relevant and the full-text articles were retrieved for a more thorough review. Based on the following reasons, 19 studies were excluded: not a relevant population (n = 6), not a relevant outcome (n = 8), and review articles (n = 5). Finally, 8 studies and 1,578 enrolled patients, were included in the meta-analysis^2,11–17^. The main characteristics of these 8 studies are summarized in Table 1 and the definition of sepsis in each study was presented in Supplemental Table S1, respectively. In all the studies included, the observational study design was used; and this meta-analysis compared the mean TH value between those who survived and those that died, in these studies.

**Table 1:** Characteristics of studies included in the review.

Identification	Location	Inclusion period	Population	Inclusion criteria	TFT^ a^	Measurement device, company	Sampling time. Day	Age, yr	Male, %	Mortality	
										%,	Time of measurement
Cui 2007	China	2004–2005	22	Severe sepsis, Septic shock	T4,T3, TSH	NR	D1, D3, D5	65.6 ± 17.7	81%	36% (8/22)	In-hospital
Gonzalez* 2016	Spain	NR	50	Severe sepsis, Septic shock	fT4, fT3	ECLIA (Roche)	D3	75.5 (59.7–80.0)	62%	30% (15/50)	In-hospital
Hosny 2015	Egypt	2013–2014	80	Sepsis, Severe sepsis, Septic shock	fT4, fT4, TSH	ELISA (BioCheck)	D5	Survivors: 46.3 ± 13.8 non-survivors: 65.7 ± 14.2	75%	48% (39/80)	In-hospital
Leo-Sanz 1997	Spain	NR	27	Septic shock	T4, T3	RIA (Behringwerke AG,Marburg)	D5	50 ± 19	66%	44% (12/27)	In-hospital
Mangas 1990	Spain	NR	37	Sepsis	T4, T3, TSH	RIA (Diagnostic Products Corporation)	During admission	57 ± 17.8	100%	40% (15/37)	In-hospital
Meyer 2011	Switzerland	11 yr	103	SIRS, Sepsis, Severe sepsis, Septic shock	T3, fT4	ECLIA (Roche)	D1, D2, Discharge day	59(46–68)	54%	23% (24/103)	In-hospital
Sumita	USA	NR	41	Sepsis	T4, T3, fT4, fT3, TSH	RIA (Dainabot)	D1	Male 31(17–77) Female 10(22–77)	75%	56% (23/41)	In-hospital
Todd 2012	USA	2 yr	231	Sepsis Severe sepsis, Septic shock	T4, T3, TSH	NR	During admission	59 ± 3	43%	17%(41/231)	In-hospital

Abbreviations: D, day of admission; ECLIA, electro-chemiluminescence immunoassay; ELISA, enzyme-linked immunosorbent assay; fT3, free triiodothyronine; fT4, free thyroxine; NR, not reported; T3, triiodothyronine; T4, thyroxine; RIA, radioimmunoassay; SIRS, systematic inflammatory response syndrome; TFT, thyroid function test; TSH, thyroid-stimulating hormone. ^*^Abstract only. ^a^T4(µg/dL), T3(ng/dl), fT4(ng/dL), fT3(pg/ml), TSH(µIU/ml). ^b^Age was presented as median (interquartile range) or mean ± standard deviation.

### Quality of the Included Studies

Among the 8 studies that were included in this present study, using the quality scoring system, 3 out of 8 studies were rated as low-quality^11,13,17^; whereas, with the remaining 5 studies, based on the scoring, the ratings were deemed to be high^2,12,14–16^. The following major factors affected the study quality: study participation/attrition as well as the study confounding. The summary of our assessment of the risk of bias of the studies included is shown in the Supplemental Fig. S1.

### Main Analysis

**Figure 2:**
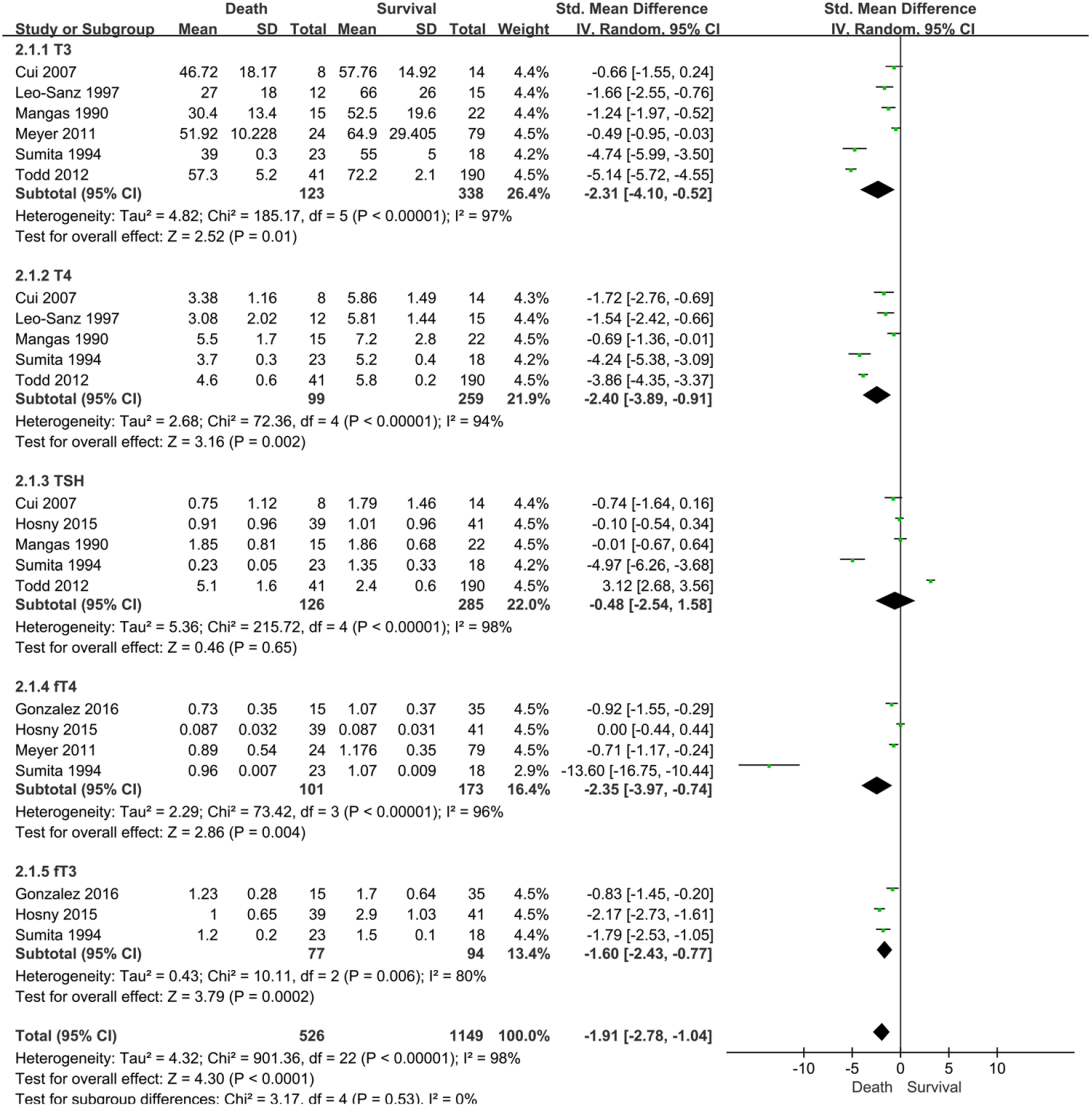
Forest plot of the effect of low thyroid hormone level and mortality before sensitivity analysis CI: confidence interval, SD: standard deviation.

Eight relevant studies, with 1,578 patients, were included in this present study. These studies reported differences according to five different serum TH levels (T3, T4, free T3, free T4, and TSH) between survivors and non-survivors (Fig. 2).

T3 levels in non-survivors were relatively lower than that of survivors (6 studies; SMD, 2.31; 95% CI, 0.52–4.10; I^2^ = 97%; P = 0.01). T4 levels in non-survivors were also lower than that of survivors with sepsis (5 studies; SMD, 2.40; 95% CI, 0.91–3.89; I^2^ = 94%; P = 0.002). There were no statistically significant differences in TSH levels between non-survivors and survivors. In the analysis for fT4 and fT3, these TH levels in non-survivors were relatively lower than that of survivors (4 studies for fT4: SMD, 2.35; 95% CI, 0.74–3.97; I^2^ = 96%; P = 0.004; 3 studies for fT3: SMD, 1.60; 95% CI, 0.77–2.40; I^2^ = 80%; P = 0.0002).

However, substantial heterogeneity was present in the data. These potential sources of heterogeneity were evaluated through additional stratified analyses (Table 2).

**Table 2:** Subgroup analysis of included studies to identify the association of thyroid hormone level with mortality.

Characteristics	Mortality, T3				Mortality, T4			
	N	T3, SMD (95% CI)	P value for heterogeneity	I^2^, %	N	T4, SMD (95% CI)	P value for heterogeneity	I^2^, %
ALL	6	−2.31(−4.10, −0.52)	P < 0.00001	97	5	−2.40(−3.89, −0.91)	<0.00001	94
**Male(%)**								
≥70	3	−2.43(−5.54, 0.68)	<0.00001	99	3	−2.18(−4.18, −0.17)	<0.00001	93
<70	3	−2.16(−4.23, −0.09)	<0.00001	93	2	−2.73(−5.01,−0.46)	<0.00001	95
**Sample size**								
≥80	2	−2.81(−7.36, 1.74)	<0.00001	99	1	−3.86(−4.35, −3.37)	—	—
<80	4	−2.01(−3.45, −0.58)	<0.00001	90	4	−2.00(−3.38, −0.62)	<0.00001	89
**Study quality**								
Low	2	−1.41(−1.97, −0.84)	0.48	0	2	−1.07(−1.90, −0.24)	0.13	56
High^*^	4	−2.74(−5.45, −0.03)	<0.00001	98	3	−3.29(−4.67, −1.92)	0.0006	87
**Mortality(%)**								
≥40	3	−2.48(−4.32, −0.64)	<0.00001	91	3	−2.11(−3.99, −0.22)	<0.00001	93
<40	3	−2.10(−5.30, 1.10)	<0.00001	99	2	−2.84(−4.49, 0.75)	0.0003	93
**Measurement techniques**								
RIA	3	−2.48(−4.32, −0.64)	<0.00001	91	3	−2.11(−3.99, −0.22)	<0.00001	93
others(2 NR/1 ECLIA)	3	−2.10(−5.30, 1.10)	<0.00001	99	2	−2.84(−4.94, −0.75)	0.0003	93

Abbreviations: 95% CI, 95% confidence interval; ECLIA, electro-chemiluminescence immunoassay; N, the number of studies; NR, not reported; RIA, radioimmunoassay; SMD, standard mean difference; T3, Triiodothyronine; T4, thyroxine. ^*^High-quality studies were those that achieved >4.5 points in quality assessment.

### Subgroup Analysis and Sources of Heterogeneity

Subgroup analysis was performed to identify the association of low T3 or T4 with mortality, and to minimize heterogeneity in each subgroup. In the analysis based on the proportion of males (≥70% vs. <70%), the total number of samples (≥80 vs. <80), mortality rate (≥40% vs. <40%), and the kinds of measurement techniques (radioimmunoassay [RIA] vs. others), there was no significant decrease in heterogeneity in each subgroup (all I^2^ > 90%). We only observed significant decrease in heterogeneity in the low quality study subgroup (T3, I^2^ = 0%, p = 0.48; T4, I^2^ = 56%, p = 0.13) (Table 2).

### Sensitivity Analysis

In the sensitivity analysis, we found two outlier studies by Sumita *et al*.^12^ and Todd *et al*.^16^, which showed significant heterogeneities between studies for T3, T4, TSH, and fT4 (Supplemental Fig. S2).

**Figure 3:**
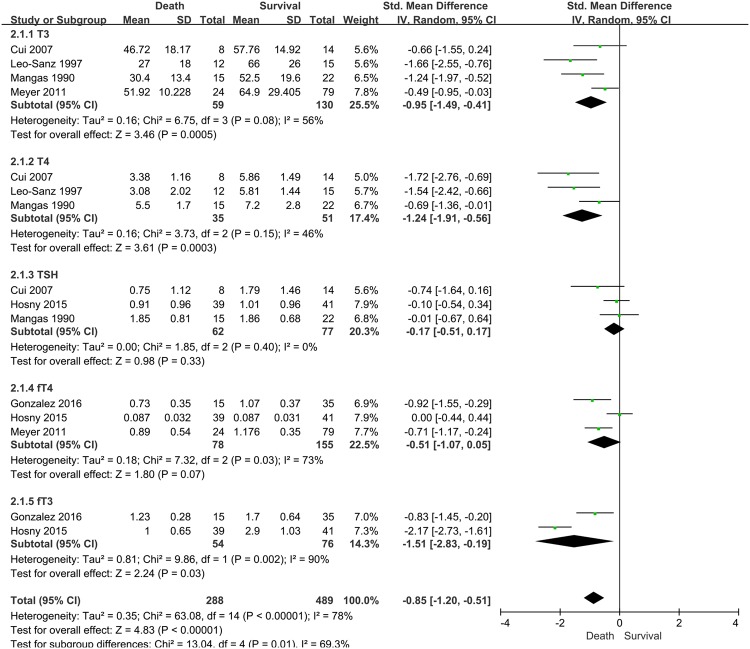
Forest plot of the effect of low thyroid hormone level and mortality after sensitivity analysis. Two outlier studies by Sumita *et al*. and Todd *et al*. were excluded following sensitivity analysis. CI: confidence interval, SD: standard deviation.

After performing the sensitivity analysis and eliminating these two studies, compared to the survivors, a significantly lower TH level was found in non-survivors; demonstrating the increase in mortality (SMD, 0.85; 95% CI, 0.51–1.20; I^2^ = 78%; P < 0.0001; Fig. 3).

T3 levels in non-survivors were relatively lower than that of survivors (4 studies; SMD, 0.95; 95% CI, 0.41–1.49; I^2^ = 56%; P = 0.0005). T4 levels in non-survivors were also lower than that of survivors with sepsis (3 studies; SMD, 1.24; 95% CI, 0.56–1.91; I^2^ = 46%; P = 0.0003). There were no statistically significant differences in TSH and fT4 levels between non-survivors and survivors. In the analysis for fT3, there were insufficient studies to perform meta-analysis.

### Publication bias

**Figure 4:**
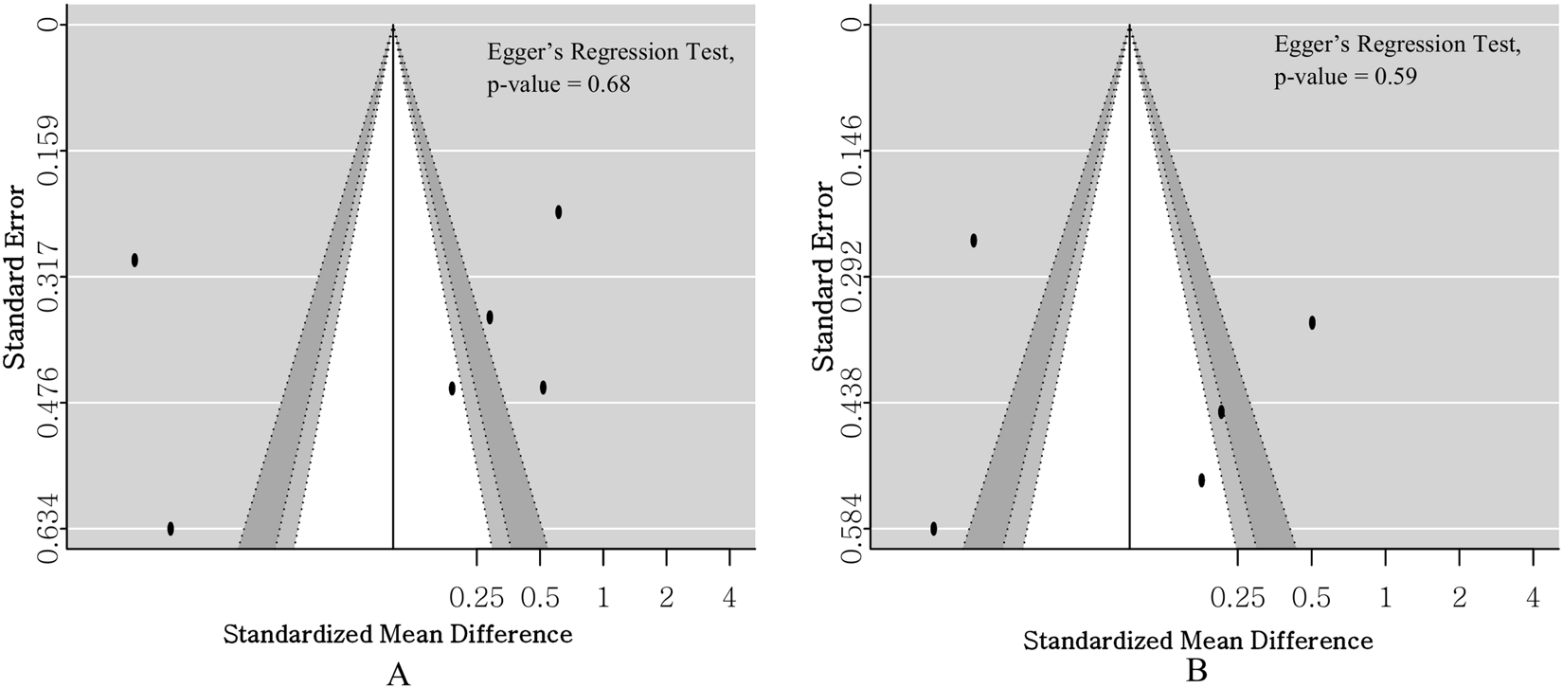
Funnel plot and Egger’s regression test to assess for publication bias. (**A**) Publication bias for low triiodothyronine (T3) level and mortality in septic patients. (**B**) Publication bias for low thyroxine (T4) level and mortality in septic patients.

There was no definite asymmetry of the forest plot. No significant bias existed statistically in the assessment based on Egger’s regression test (T3, p = 0.68; T4, p = 0.59) (Fig. 4).

## Discussion

According to the findings of this systematic review and meta-analysis, decreased TH levels, either T3 or T4, tend to be associated with mortality in adult septic patients. These results suggest the possibility of decreased thyroid level (T3 or T4) as a prognostic factor of mortality in adult septic patients. As far as we know, this meta-analysis is the first to evaluate the value of TH as a prognostic factor, in a total 1,578 adult patients with sepsis.

Recent systematic review reported that lower T3 or T4 showed associations with worse outcome in sepsis or septic shock^9^. However, only two included studies were in adults^11,13^ while most studies were among neonates and children. In addition, higher serum TH levels in neonates were also shown in comparison with those of older children and adults; these results were indicative of adult septic patients^9^. Therefore, we aimed at a meta-analysis that was restrictively performed only in adult patients with sepsis.

The main mechanism of sepsis may involve systematic inflammatory effects, immune dysfunction, the dysfunction of blood coagulation and anti-coagulation, and tissue injury^18–20^. It has been shown that critical illnesses including sepsis was often accompanied by alterations in the levels or function of several hormones. Changes in the level of serum THs without concomitant thyroid disease are known as non-thyroidal illness syndrome (NTIS). NTIS in sepsis is mainly caused by acute severe inflammation, which can impair the function of the hypothalamic-pituitary-thyroid axis^21,22^. TH may therefore significantly decrease as sepsis progresses, which promotes multiple organ dysfunctions in septic patients^23,24^.

TH is closely related to the immune system, which could influence the function and activity of neutrophils and other immune cells. As a result, decreased TH level could cause immune system dysfunction^25–27^.

With the coagulation system, anti-thrombin III plays an important role in maintaining the balance between coagulatory and anticoagulatory functions in septic patients. A previous study reported that T3 supplement could protect septic patients from disseminated intravascular coagulopathy (DIC) through increasing the levels of anti-thrombin III^10^. Hence, decreased TH could worsen the prognosis and increase the mortality in septic patients.

In the cardiovascular system, cardiac dysfunction is observed in septic patients^28^. The severity of cardiac dysfunction was shown to be correlated with the prognosis of sepsis^28–31^. The appropriate level of THs is essential for the maintenance of the normal electrical activity of the heart muscle^5^. Therefore, disorder in TH may favor septic cardiac dysfunction and influence the prognosis in septic patients^24,32,33^.

THs are also associated with respiratory functions. Increased serum T3 levels can augment the synthesis of pulmonary surfactant, reduce alveolar surface tension, and increase the lung compliance; resulting in an improved lung function^6,7^. Decreased level of pulmonary surfactant has been found in septic patients, which led to septic pulmonary dysfunction^34^.

These significant correlations between decreased TH and multiple organ dysfunctions indicate the possibility of decreased TH as another prognostic factor of mortality in sepsis. This is because the main pathophysiology of death in sepsis patient involves multiple organ dysfunction syndromes^35^. Several studies have also demonstrated that the decrease in TH level was clinically associated with the increase in mortality in adult septic patients^2,11–17^.

The point in time window measurement of TH level could be altered in septic patient according to the progression of sepsis. In the early phase of sepsis, alterations in TH involve mainly peripheral mechanisms, such as, impaired peripheral conversion of T4 to T3 resulting in low T3^21,36^. During the late phase of sepsis, alteration in TH is associated with centrally induced hypothyroidism leading to low T4 and low-normal or decreased TSH in addition to the low T3^21,36,37^. Similarly, our meta-analysis also showed lower T3 or T4 in death groups among septic patients than survivors. Thus, the serial measurement of T3 and T4 in sepsis could probably be more informative to evaluate the prognosis in sepsis.

We found high heterogeneities in the statistical results of this meta-analysis. To resolve the high heterogeneity issues, subgroup analysis was first performed. Although a stratified analysis was performed for confounding factors such as sex (male ≥70% vs. <70%), sample size (≥80 vs. <80), study quality (high vs. low), and mortality (≥40% vs. <40%), the heterogeneities still remained high (Table 2). The most likely cause of heterogeneities was considered as follows: the severity of sepsis (Acute Physiology and Chronic Health Evaluation [APACHE] II, Sequential Organ Failure Assessment [SOFA], and the Simplified Acute Physiology Score [SAPS] II); the diversity of infectious focus (lung vs. gastrointestinal vs. genitourinary tract), and the inconsistent TH sampling time. These many factors which increase heterogeneities were not adjusted for because of insufficient data (Table 1 and Supplemental Table S2).

After the subgroup analysis, any substantial reduction in heterogeneities was not observed. To resolve the issue of the high heterogeneities, we additionally performed sensitivity analysis for two specific included studies^12,16^. The study by Sumita *et al*.^12^ presented the highest mortality among the included studies (56% in-hospital mortality; Table 1). Since the high mortality group in septic patients could cause relatively lower TH levels compared with the low mortality groups, this study was considered to be the major contributor of the high heterogeneity. Furthermore, the study by Todd *et al*.^16^ was also considered as another contributor, because this study was only performed in surgical septic patients. We expected that the relatively different patient characteristics of this study also caused considerable heterogeneities. After performing sensitivity analysis by removing these two studies, the heterogeneities for TH (T3, T4, and TSH) in this meta-analysis effectively reduced (T3, I^2^ = 56%; T4, I^2 ^  = 46%; TSH, I^2 ^  = 0%; Fig. 3). Nevertheless, the heterogeneities of other THs (fT3 and fT4) were not resolved after the sensitivity analysis.

This meta-analysis has several limitations. First, the severity scores in septic patients affected the prognosis and were insufficiently reflected in this meta-analysis (Supplemental Table S2). These resulted in the lack of data for the reported severity scores of included studies. Second, the high heterogeneity issues due to the inconsistent sampling time of serum TH were not sufficiently resolved. This was because only 4 of 8 included studies exactly reported the sampling time of TH (Table 1). We therefore could not examine the sampling time of the serum TH analysis. Third, general representativeness was not established in this study. Although various races such as Asian, Caucasian, and African were included, all included studies were single-center investigations. Hence, this study may not be generalized to most patients with sepsis.

In conclusion, the findings of this systematic review and meta-analysis showed that decreased serum T3 or T4 levels tend to be associated with the mortality in adult septic patients. On the basis of these findings, the measurement of serum T3 or T4 levels in adult septic patients could provide better information-related prognosis.

## Methods

Our study was based on the principles outlined by the Meta-analysis of Observational Studies in Epidemiology (MOOSE)^38^ and the Preferred Reporting Items for Systematic Reviews and Meta-analysis (PRISMA) groups^39^.

Briefly, we developed a question based on population, intervention, comparison, and outcome (PICO). Accordingly, literature searches and critical assessments were performed. We summarized the eligible studies; and their outcomes were evaluated in a meta-analysis.

The PICO question was as follows: population (P) = hadult septic patients who neither had thyroid diseases nor medication history of intake of drugs altering thyroid function; intervention (I) = decreased thyroid function (the decrease in thyroid hormone level), comparator (C) = none; outcome (O) = mortality.

### Search Strategy

A literature search was performed by two experienced reviewers (J. Kim and H. Shin) on 08 August 2017. The search encompassed the MEDLINE and EMBASE databases via the Ovid interface.

Search terms included “sepsis” or “septic shock” or “systemic inflammatory response syndrome” or “septicemia” or “bacteremia” and “thyroid hormone” or “thyrotropin” or “hypothyroidism” or “triiodothyronine” or “T3” or “thyroxine” or “T4” or “TSH” (Supplemental Table S3).

We included articles reporting any prospective or retrospective cohort studies which addressed our PICO question.

### Study Selection

All identified studies were inputted into the reference management software Endnote X8. The two reviewers checked the title, abstract, or type of each of the identified articles.

Studies were included in this meta-analysis if they met the following eligibility criteria: (1) included adult septic patients who neither had thyroid diseases nor medication history of intake of drugs altering thyroid function; (2) measured thyroid hormone levels (T3 or T4 or TSH or fT3, or fT4) during admission; (3) provided the mortality data of patients during admission or after discharge. We excluded articles based on the following exclusion criteria: reviews, case reports, editorials, letters, comments; animal studies; duplicate studies; studies on pediatric populations including adolescents, children, infants, and neonates (Fig. 1).

In case of disagreement between the two reviewers, a third reviewer (W. Kim), intervened, and differences were discussed until a consensus was reached. After eliminating the excluded abstracts, we retrieved the full-texts of the chosen articles, which were then rescreened and evaluated more thoroughly for eligibility using the same exclusion criteria. Ultimately, our selected studies included adult patients (age 18–75 years) with sepsis whose mortality and TH assay information were available.

### Data Extraction

The two reviewers extracted the characteristics and results of selected studies (J. Kim and H. Shin). Any unresolved disagreement after the discussion was further reviewed by the other co-author (W. Kim).

We extracted the following variables from the studies: first author, year of publication, country, study population, inclusion period, assay method for thyroid function test (TFT) detection, sampling time, baseline characteristics of the patients including age, sex, and in-hospital mortality, and the mean TH (T3, T4, free T3, free T4, and TSH) with their standard deviations (SDs) or the median and interquartile ranges (IQR) were used.

Where necessary, variables listed above that were not described in the studies, were requested for, from each study corresponding author via email. The corresponding author (W. Kim) for the present study had full access to all the data in the study and took responsibility for its integrity and for the data analysis.

### Risk of Bias in Individual Studies

To determine the methodological quality of the studies included, BH. Jang and TH. Lim, who were blinded to the authorship or the journal names of selected articles, assessed the studies independently. The evaluation of the risk of bias was carried out using the Quality in Prognosis Studies (QUIPS) tool, and reported as 1 (low), 0.5 (unclear), and 0 (high)^40^. The scores of the 6 items assessed were summed up to determine studies achieving >4.5 points (high quality). Reviewers of the present study resolved any disagreements through discussions, or a third author conducted a separate review.

### Statistical Analysis

The main analysis consisted of an investigation into the association between decreased thyroid function and in-hospital mortality in adult septic patients.

To determine the strength of the association between decreased thyroid function and death, the standardized mean differences (SMD) was derived between the survivors and non-survivor groups, using a random-effect model^41^.

The effect size estimation was performed by determining the SMD with their 95% confidence interval (CI) because the unit weight of TH levels were reported in most studies with a wide variety of units. SMD was calculated by the following equation: [(non-survivor group mean level − survivor group mean level)/pooled SD]. The mean difference ± SD were derived from the TH levels across comparison groups.

Various units of TH were used in our study; hence, we converted all these different TH units into a single unit: T4(µg/dl), T3(ng/dl), Free T4(ng/dl), Free T3(pg/ml), TSH(µIU/ml).

In studies without SD, the variance was derived as follows^42^: SD  Sstandard error (SE) × sqrt{N, sample size}, SD N, 1.35/IQR. Heterogeneity was estimated by determining the proportion of inconsistency resulting from the true differences between studies (rather than the differences resulting from the random error or chance) using the I^2^ statistic reported as 25% (low), 50% (moderate), and 75% (high)^43^.

We conducted a planned subgroup analyses on extracted subgroup variables for the proportions of males (≥70% vs. <70%), the sample sizes (≥80 vs. <80), and mortality rates (≥40% vs. <40%). We used the median value of the reported rates or the number of the 8 studies included as the reference, to classify both groups of each variable. The analyses of other subgroups such as study quality (low vs. high) or the measurement techniques (RIA vs. others), were also performed.

In the main and subgroup analysis, we used RevMan version 5.3 (Cochrane Collaboration, Oxford, UK) for the statistical analysis, and P < 0.05 was considered statistically significant. Random effects model was used to synthesize the individual data of included studies considering the diversity of countries, medical systems, and inclusion periods^44–49^.

Sensitivity analysis and the identification of publication bias were also performed by the R packages ‘meta’ (R version 3.3.2). Sensitivity analysis was done by sequentially omitting individual study. Publication bias was assessed by funnel plot and Egger’s test. The asymmetry of the funnel plot and P-value (<0.05) using Egger’s test indicated that bias existed.

## Electronic supplementary material

Supplemental Figures and Tables
