# Supplementary material for: The Value of Decreased Thyroid Hormone for Predicting Mortality in Adult Septic Patients: A Systematic Review and Meta-Analysis
Source: Sci Rep. 2018 Sep 20;8:14137. doi: 10.1038/s41598-018-32543-7 (PMC6148249; doi:10.1038/s41598-018-32543-7)
Supplement: Supplementary file 1 — Supplemental Figures and Tables [file 41598_2018_32543_MOESM1_ESM.pdf]

The Value of Decreased Thyroid Hormone for Predicting Mortality in Adult Septic Patients: A Systematic Review and Meta-Analysis

Jae Guk Kim, M.D.[1,†], Hyun Goo Shin, M.D.[2,†], Wonhee Kim, M.D.[1,3,*], Tae Ho Lim, M.D., PhD.[2], Bohyoung Jang, M.D.,Ph.D.,M.P.H[5], Youngsuk Cho, M.D.[1,3], Kyu-Sun Choi, M.D.[4], Chiwon Ahn, M.D.[3,6], Juncheol Lee, M.D.[2], Min Kyun Na, M.D.[4]

[1]Department of Emergency Medicine, College of Medicine, Hallym University, Chuncheon, Republic of Korea

[2]Department of Emergency Medicine, College of Medicine, Hanyang University, Seoul, Korea

[3]Department of Biomedical Engineering, Graduate School of Medicine, Hanyang University, Seoul, Korea

[4]Department of Neurosurgery, College of Medicine, Hanyang University, Seoul, Korea

[5]Department of Preventive Medicine, College of Korean Medicine, Kyung Hee University, Seoul, Korea

[6]Department of Emergency Medicine, Armed Forces Yangju Hospital, Yangju, Korea

[†]Kim and Shin contributed equally to this work.

[*]**Corresponding Author**: Wonhee Kim, M.D.

1, Singil-ro, Yeongdeungpo-gu, Seoul, 07441, Republic of Korea

Tel: +82-2-829-5561

Fax: +82-2-842-4217

E-mail: wonsee02@gmail.com

Key Words: Thyroxine (T4); Triiodothyronine (T3); Thyroid-stimulating hormone; Sepsis; Mortality; Meta-Analysis

(A)

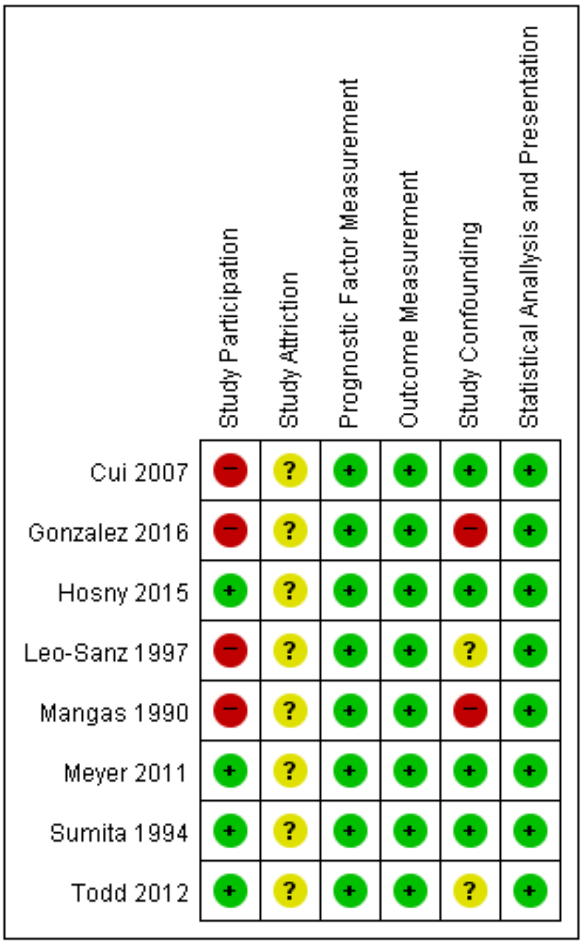

(B)

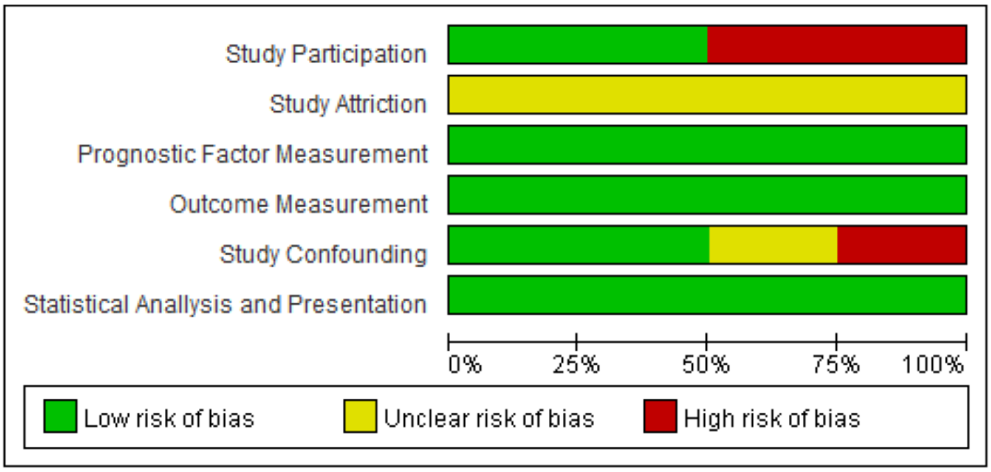

Supplemental Figure S1. The summary of assessment of the risk of bias of the included studies

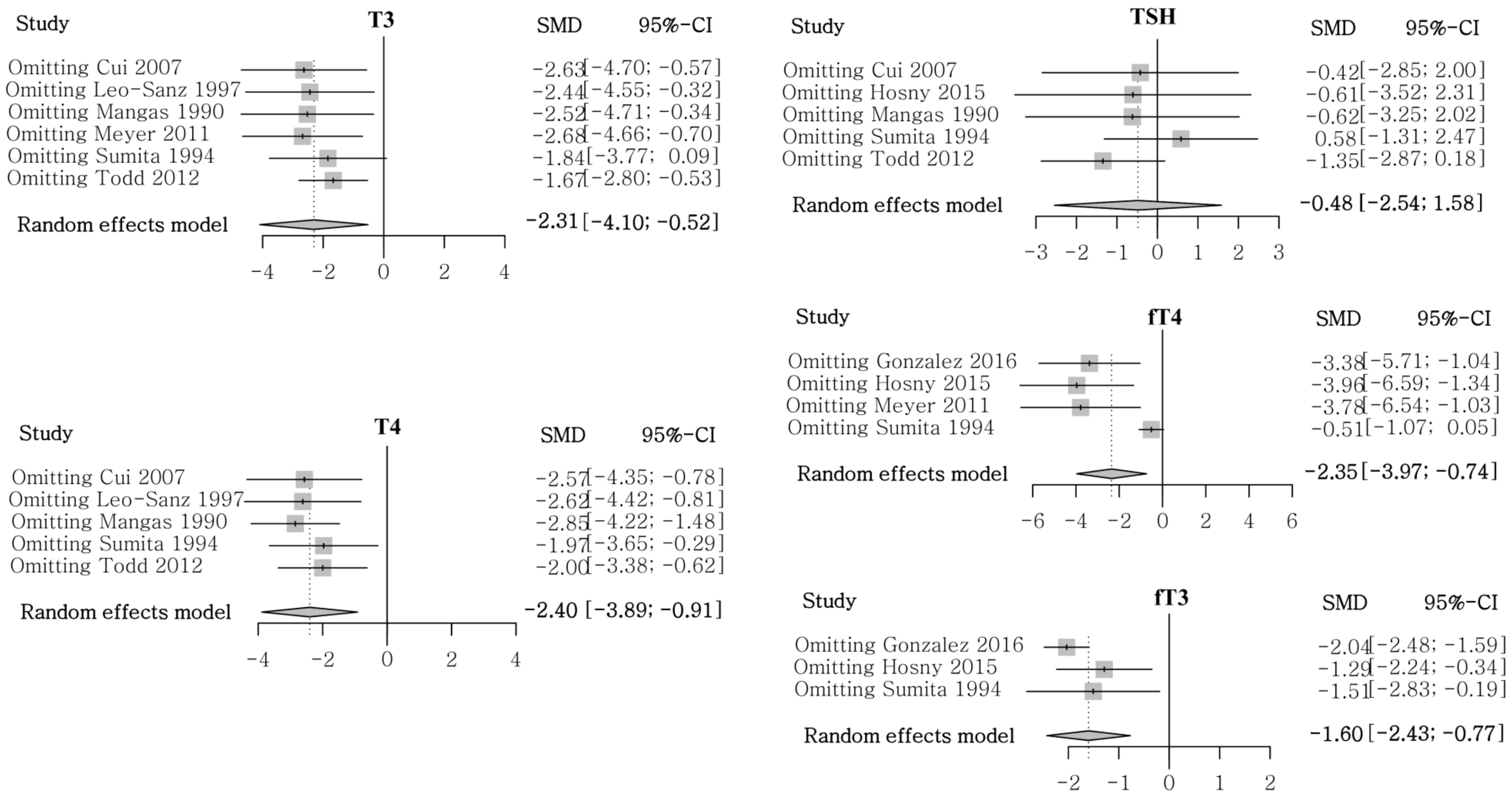

Supplemental Figure S2. The sensitivity analysis of the included studies

Supplemental Table S1. Definition of sepsis

Identification	Inclusion criteria	Definition of SIRS, sepsis, severe sepsis, and septic shock
Cui 2007	Severe sepsis,<br>Septic shock	Defined by the criteria of ACCP/SCCM in 1992:<br><br>Severe sepsis is the sepsis associated with organ dysfunction, hypoperfusion, or hypotension. Hypoperfusion and perfusion abnormalities may include, but are not limited to lactic acidosis, oliguria, or an acute alteration in mental status.<br><br>Septic shock is the sepsis-induced with hypotension despite adequate fluid resuscitation along with the presence of perfusion abnormalities that may include, but are not limited to, lactic acidosis, oliguria, or an acute alteration in mental status. Patients who are receiving inotropic or vasopressor agents may not be hypotensive at the time that perfusion abnormalities are measured.
Gonzalez 2016	Severe sepsis,<br>Septic shock	Severe sepsis or septic shock (abstract only).
Hosny 2015	Sepsis,<br>Severe sepsis,<br>Septic shock	Sepsis is a culture-proven or visually identified infection accompanied by two or more of the following abnormalities (temperature >38.5 ℃ or <36 ℃, heart rate >90 beats/min, respiratory rate >20 breaths/min or PaCO2 <32 mmHg and TLC count >12,000 cells/mm3, <4000 cells/mm3, or >10 percent immature (band) forms.<br><br>Severe sepsis group included patients with sepsis accompanied by organ failure or hypoperfusion including oliguria and altered mental status.<br><br>Septic shock group included patients with severe sepsis accompanied by hypotension (systolic blood pressure <90 mmHg or a fall off >40 mmHg from the baseline in the absence of other causes of hypotension).
Leo-Sanz 1997	Septic shock	Defined by the criteria of ACCP/SCCM in 1992:<br><br>Septic shock is a sepsis-induced with hypotension despite adequate fluid resuscitation along with the presence of perfusion abnormalities that may include, but are not limited to, lactic acidosis, oliguria, or an acute alteration in mental status. Patients who are receiving inotropic or vasopressor agents may not be hypotensive at the time that perfusion abnormalities are measured.
Mangas 1990	Sepsis	Fever above 38℃, characteristic clinical signs, and positive in blood cultures with the same microorganism.
Meyer 2011	SIRS,	Defined by the criteria of ACCP/SCCM in 1992:

	Sepsis,<br>Severe sepsis,<br>Septic shock	SIRS is the systemic inflammatory response to a variety of severe clinical insults. The response is manifested by two or more of the following conditions: (I) temperature >38℃ or <36℃; (2) heart rate >90 beats per minute; (3) respiratory rate >20 breaths per minute or PaC02 <32 mm Hg; and (4) white blood cell count>12,000 /cu mm, <4,000/cu mm, or > 10% immature (band) forms.<br><br>Sepsis is the systemic response to infection, manifested by two or more of the following conditions as a result of infection: (1) temperature >38℃ or <36℃; (2) heart rate >90 beats per minute; (3) respiratory rate >20 breaths per minute or PaC02 <32 mm Hg; and white blood cell count > 12,000 /cu mm, <4,000/cu mm, or > 10% immature (band) forms.<br><br>Severe sepsis is the sepsis associated with organ dysfunction, hypoperfusion, or hypotension. Hypoperfusion and perfusion abnormalities may include, but are not limited to lactic acidosis, oliguria, or an acute alteration in mental status.<br>Septic shock = sepsis-induced with hypotension despite adequate fluid resuscitation along with the presence of perfusion abnormalities that may include, but are not limited to, lactic acidosis, oliguria, or an acute alteration in mental status. Patients who are receiving inotropic or vasopressor agents may not be hypotensive at the time that perfusion abnormalities are measured.
Sumita 1994	Sepsis	Sepsis was defined by septic score. The septic score is a simple system for grading the severity of sepsis has been developed by scoring the attributes of sepsis under four headings: local effects of infection, pyrexia, secondary effects of sepsis and laboratory data.
Todd 2012	Sepsis (surgical)<br>Severe sepsis,<br>Septic shock	Surgical sepsis is defined as the systemic inflammatory response syndrome in addition to an infection requiring surgical intervention for source control or systemic inflammatory response syndrome plus an infection within 14 days of a major surgical procedure.

SIRS = severe inflammatory response syndrome, ACCP = American College of Chest Physicians, SCCM = Society of Critical Care Medicine

Supplemental Table S2. Detailed information of population

Study identification	Infection site			Severity			ICU stay
	Lung, %	Gastrointestinal tract,%	Genitorurianry tract, %	APACHE II , Mean ±SD	SOFA score , Mean ±SD	SAPS II Median(IQR)	Day Mean ±SD or Median(IQR)
Cui 2007	-	-	-	-			-
Gonzalez[A] 2016	-	-	-				-
Hosny 2015	41.5 / 53.8	14.6 / 35.9	23.3 / 12.8	10.3 ± 3.2 / 26.6 ± 7	D2: 2.7 ± 1.6 / 14±2.8<br>D5: 0.9 ± 0.9 / 17.4±3		8.2±4.4 / 21.4±18.8
Leo-Sanz 1997	53.3 / 41.6	26.6 / 50	20 / 8.3				-
Mangas 1990							-
Meyer 2011	52.8 / 64.7	22.2 / 23.5	19.4 / 11.8	21(16-25.5) / 27(19-31.8)		50(39-62) / 67(53-83)	3 (1-4)†
Sumita 1994	-	-	-	15.7 ± 1.2 / 21.7 ± 1.6			-
Todd 2012	-	-	-	-			-

Each domain is described as a value corresponding to survivors / non-survivors

[*]These are the results of the total population including survivors and nonsurvivors.

[†]The value was measured for both survivors and non-survivors.

Supplemental Table S3. Comprehensive list presenting the search strategy

Sepsis_Thyroid

EMBASE 1806

MEDLINE 316

EMBASE

1. exp systemic inflammatory response syndrome/	225028
2. septic shock/ or sepsis/ or severe sepsis.mp.	161635
3. exp bacteremia/ or bacteremia.mp.	49919
4. bacteraemia.mp.	7186
5. septicemia/ or septicemia.mp.	26450
6. septicaemia.mp.	7041
7. 1 or 2 or 3 or 4	240671
8. exp thyroid hormone/	30047
9. exp thyroxine/	51604
10. triiodothyronine.mp.	18581
11. exp hypothyroidism/	59781
12. exp thyrotropin/	56319
13. T4.mp.	56996
14. T3.mp.	57919
15. exp thyroid function/	17170
16. exp hyperthyroidism/	59848

17. TSH.mp.	41052
18. 8 or 9 or 10 or 11 or 12 or 13 or 14 or 15 or 16 or 17	247168
19. 7 and 18	1806

Medline

1. exp Systemic Inflammatory Response Syndrome/	113790
2. exp Sepsis/	110232
3. exp Shock, Septic/	20714
4. severe sepsis.mp.	7689
5. exp Bacteremia/	26166
6. bacteraemia.mp.	5420
7. septicemia.mp.	14028
8. septicaemia.mp.	6104
9. 1 or 2 or 3 or 4 or 5 or 6	127460
10. exp Thyroid Hormones/	62598
11. exp Triiodothyronine/ or exp Hypothyroidism/ or exp Thyroxine/ or exp Thyrotropin/	85540
12. T4.mp.	43086
13. T3.mp.	40983
14. thyroid function.mp.	25001
15. exp Hyperthyroidism/	42494
16. TSH.mp.	28879
17. 10 or 11 or 12 or 13 or 14 or 15 or 16	182587
18. 9 and 17	316
